# FRET-FLIM Investigation of PSD95-NMDA Receptor Interaction in Dendritic Spines; Control by Calpain, CaMKII and Src Family Kinase

**DOI:** 10.1371/journal.pone.0112170

**Published:** 2014-11-13

**Authors:** Kim Doré, Simon Labrecque, Christian Tardif, Paul De Koninck

**Affiliations:** 1 Institut Universitaire en Santé Mentale de Québec, Université Laval, Québec, QC, Canada; 2 Département de Biochimie, Microbiologie et Bio-informatique, Université Laval, Québec, QC, Canada; Institute for Interdisciplinary Neuroscience, France

## Abstract

Little is known about the changes in protein interactions inside synapses during synaptic remodeling, as their live monitoring in spines has been limited. We used a FRET-FLIM approach in developing cultured rat hippocampal neurons expressing fluorescently tagged NMDA receptor (NMDAR) and PSD95, two essential proteins in synaptic plasticity, to examine the regulation of their interaction. NMDAR stimulation caused a transient decrease in FRET between the NMDAR and PSD95 in spines of young and mature neurons. The activity of both CaMKII and calpain were essential for this effect in both developmental stages. Meanwhile, inhibition of Src family kinase (SFK) had opposing impacts on this decrease in FRET in young versus mature neurons. Our data suggest concerted roles for CaMKII, SFK and calpain activity in regulating activity-dependent separation of PSD95 from GluN2A or GluN2B. Finally, we found that calpain inhibition reduced spine growth that was caused by NMDAR activity, supporting the hypothesis that PSD95-NMDAR separation is implicated in synaptic remodeling.

## Introduction

The dynamic molecular reorganization that takes place in dendritic spines is under increasing investigation, as it supports synaptic remodeling, a process required for memory formation. A prime influence on synaptic remodeling is the activity of the synapse and the activation of the NMDA receptor (NMDAR). Calcium influx through NMDAR is a fundamental triggering signal that sets in motion a series of biochemical cascades and protein re-distribution and interactions, leading to long-term changes in synaptic transmission [1], [2]. The cytoplasmic tails (c-tails) of the NMDAR are central hubs where several signaling proteins, such as kinases and phosphatases, and anchoring proteins, including PSD95, converge [3]. Some of these kinases, namely CaMKII and Src, phosphorylate GluN2 subunits or PSD95 particularly during synaptic plasticity [4], [5]. Another process that concerns active GluN2 subunits of the NMDAR is the intervention of calpain, which can cleave their c-tails, under the regulation of kinases and PSD95 [6]–[9]. However, such cleaving function at synaptic NMDARs and its role in synaptic plasticity have not been established. The changing ratio of GluN2A/2B during post-natal development [10]–[13] likely impacts on the involvement of calpain and these kinases in NMDAR signaling [14].

The molecular dissection of these signaling steps inside spines and determining their implication in synaptic development and plasticity are challenging tasks, given the limiting spatial and temporal resolution of most available methods. Using two-photon imaging and glutamate uncaging, Steiner et al. have been able to establish a link between the transient exit of PSD95 from a spine and its remodeling [5]. A possible mechanism underlying this process would involve an activity-dependent dissociation of PSD95 from the NMDAR. Given the critical role that this interaction is thought to play in synaptic plasticity [5], [15], [16] and in synaptic dysfunction and excitotoxicity [17], [18], we established a method to quantify PSD95-NMDAR interaction in living spines undergoing activity-dependent remodeling.

We used Fluorescence Lifetime Imaging (FLIM) to quantify Förster Resonance Energy Transfer (FRET) between NMDARs and PSD95 labeled with fluorescent proteins, as a proxy for direct protein-protein interaction. We demonstrate the activity-dependent separation of PSD95 from the NMDAR, as well as the roles of CaMKII, calpain and SFK in differentially controlling this dissociation during synaptic maturation. We also show a correlative change in calpain-dependent spine growth and separation of PSD95 from the NMDAR in response to NMDAR stimulation. Our work sheds new light on the molecular reorganization that takes place inside spines undergoing remodeling and introduces a method that will help the further dissection of intra-spine molecular signaling during development, plasticity or destructive processes.

## Materials and Methods

### Rat hippocampal cultures, plasmids and transfection

Dissociated hippocampal neurons were prepared as described [19], [20]. In brief, hippocampi were dissected out of P1-P3 rats dissociated enzymatically (papain, 12 U/ml; Worthington) and mechanically (trituration through Pasteur pipette). Rats pups were decapitated and tissue was harvested according to protocol approved by the animal protection comity of Laval University; CPAUL (Comité de Protection des animaux de l'Université Laval). After dissociation, the cells were washed, centrifuged, and plated on poly-D-lysine–coated Aclar (12 mm) or glass (18 mm) coverslips at a density of 350–450 cells/mm^2^. Neurons were then transfected at DIV7, DIV14 or DIV21 using Lipofectamine 2000 (Invitrogen, San Diego, CA) as described previously [19]. GluN1-mGFP plasmid was generated by replacing GFP from GluN1-GFP (obtained from A.M. Craig [21]) with monomeric GFP (to eliminate any possible dimerization of GFP). PSD95-mCherry was generated by replacing mGFP with mCherry [22]. PSD95-S73A-mCherry and PSD95-S73D-mCherry were generated by point mutation of PSD95-mCherry.

### Imaging solutions and fixation

The standard imaging solution consisted of Hank's Balanced Salt Solution (HBSS without Ca^2+^, Mg^2+^, bicarbonate, Invitrogen) supplemented with (in mM) 10 HEPES, and 2.0 glucose. To maintain little or no NMDAR activity, this solution contained also 0.6 CaCl_2_ and 5.0 MgCl_2_ [19]. For glutamate/glycine (Glu/Gly) stimulation, this HBSS solution contained instead 1.2 CaCl_2_, 1.0 MgCl_2_, 0.1 glutamate and 0.01 glycine (VWR). For chemical LTP stimulation (0Mg^2+^/Gly), the HBSS solution contained 1.2 CaCl_2_ and 0.2 Glycine. The osmolality of all solutions was adjusted to 260 mOsm (to match that of the Neurobasal media) and pH was adjusted to 7.3. AP5, KN92, KN93, PP2, PP3, PD150606 and ionomycin were from Calbiochem, MDL-28170 was from Tocris and MK-801 from VWR. When specified, AP5 was applied only during the 1 min Glu/Gly stimulation. Neurons were pre-incubated in the culture medium with KN92, KN93, PP2, PP3, PD150606, MDL-28170 and MK-801 for 1 hour before the experiment and co-applied with stimulation using the concentrations specified in the text. Where specified, hippocampal neurons were fixed before imaging, using methanol (−20°C) for 10 min. After fixation, cells were rinsed twice with PBS and coverslips were mounted in Prolong Gold (Invitrogen).

### FRET/FLIM and confocal imaging

FRET-FLIM was used with both living and methanol-fixed neurons. For the latter approach, we characterized the effect of fixing and mounting cells on GFP lifetime using HEK cells transfected with GluN1-GFP and untagged GluN2B. We observed that in fixed cells, the lifetime of GFP was reduced from 2.493±0.009 ns (N = 15 cells) to 2.249±0.009 ns (N = 22). This effect is due to the higher refractive index of the Prolong Gold mounting solution (n = 1.46), as reported earlier [23]. However, we found that the lifetime change induced by this procedure (≈240 ps) was constant if the time allowed for curing was sufficient (≥2 days) and if the samples were imaged within a month from fixation/mounting (Figure S2A, in file S1). Thus, the reproducibility of this approach allowed the combination of data from different culture preparations over months. Moreover, we verified whether fixation and mounting had an impact on the FRET efficiency observed between GluN1-GFP and PSD95-mCherry. To do so, we expressed GluN1-GFP/GluN2B/PSD95-mCherry or GluN1-GFP/GluN2B in HEK cells and measured the GFP lifetime before and after fixation and mounting. FRET efficiency (EFRET) was calculated for each condition using the following formula: EFRET =  1- (τ_DA_/τ_D_) (τ_DA_ is the lifetime of the FRET donor, GluN1-GFP, in the presence of a FRET acceptor; τ_D_ is the lifetime of GluN1-GFP expressed alone). We found no difference in the FRET efficiency between live (EFRET =  6.3±0.4, N = 67) and fixed cells mounted in Prolong Gold (EFRET =  6.6±0.3, N = 42) (unpaired t-test p = 0.46) (Figure S2C in file S1). Broussard et al [24] reported a reduced FRET efficiency of their sensors upon mounting cells in Prolong Gold, suggesting that these effects may depend on the probes and fixation method used (e.g. methanol vs paraformaldehyde).

**Figure 1:**
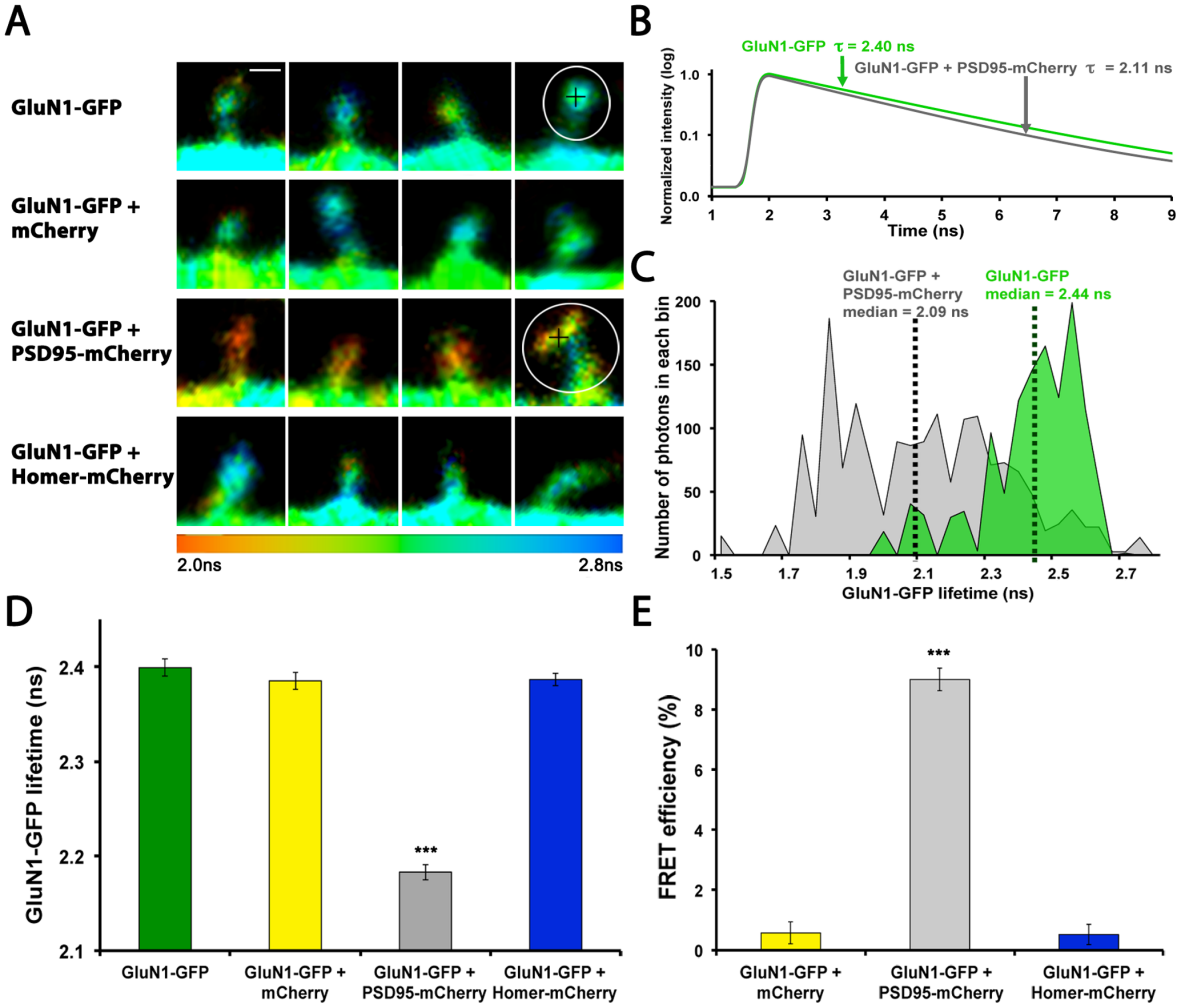
GluN1-GFP and PSD95-mCherry are specific probes for measuring the interaction between PSD95 and the NMDAR with FRET-FLIM. (A) FLIM images of GluN1-GFP expressing spines, expressed alone (top row), with mCherry (second row), with PSD95-mCherry (third row) or Homer-mCherry (last row). Scale bar is 1 µm (placed in first spine). Color coding represents GluN1-GFP lifetime from 2 ns to 2.8 ns. Black crosses indicate the pixel selected for traces in B. Circles indicate ROI for panel C. (B) Fit curves of fluorescence intensity decays obtained from one pixel (black crosses in the spines shown in A) for a GluN1-GFP expressing spine (green curve) and GluN1-GFP/PSD95-mCherry expressing spine (black curve), see Fig. S1 in file S1 for raw data. (C) Distribution histograms of GluN1-GFP lifetimes in encircled spines in A (GluN1-GFP, green; GluN1-GFP/PSD95-mCherry, gray), the median (dotted line) of each distribution was averaged across samples to produce a mean lifetime. (D) The mean lifetime in GluN1-GFP/PSD95-mCherry expressing spines (14 neurons (N)/258 spines (s), gray) is significantly shorter than GluN1-GFP alone, indicating FRET between the NMDAR and PSD95. Spines expressing either GluN1-GFP (12 N/160 s, green), GluN1-GFP/mCherry (12 N/123 s, yellow) or GluN1-GFP/Homer-mCherry (13 N/182 s, blue) all have similar lifetimes. Statistical analysis was performed by Kruskal-Wallis test (p<0.0001) followed by Dunn's test. GluN1-GFP/PSD95-mCherry group is different from all the other conditions p<0.001. (E) FRET efficiency in the same spines as in D. Kruskal-Wallis test was performed (p<0.0001), followed by Dunn's test. GluN1-GFP/PSD95-mCherry group is different from all the other conditions p<0.001.

Neuronal cultures were illuminated with a Chameleon Ultra IR laser (Coherent) at 80 MHz repetition rate tuned at 900 nm for GFP two-photon excitation. Fluorescence emission was detected with a cooled high speed PMT detector head (PMC-100-1, Becker and Hickl, Germany) between 505–545 nm by means of a GFP emission filter (510/42 nm BrightLine single-band bandpass filter, Semrock) coupled to a laser block filter (750 nm blocking edge BrightLine multiphoton short-pass emission filter). The acquisition of fluorescence lifetimes was synchronized by a time-correlated-single-photon-counting (TCSPC) module (SPC-830, Becker and Hickl, Germany). Measurements were performed on a Zeiss LSM 510 microscope using a 40x water immersion objective (Achroplan, Zeiss) for live experiments and a 60x water immersion objective (Olympus UPLSAPO 60XW, NA = 1.2) for fixed samples. The following parameters were kept constant for all acquired images: pixel size (90 nm; all 512×512 pixels) (a pixel size of 45 nm was used only for figure 1), pixel dwell time (1.6 µs), laser excitation intensity (a maximum of 2 mW after the microscope objective), and FLIM acquisition time (30–60 seconds/image). Reference green and red images in confocal mode were also recorded for each FLIM image (GFP excitation 488 nm, detection through a band-pass filter (500–530 nm), mCherry excitation 543 nm, detection through a band-pass filter (565–615 nm)).

### FLIM analysis

Fluorescence lifetime images were analyzed with SPCimage (Becker and Hickl). For FLIM measurements in individual dendritic spines, we typically collected an insufficient amount of photons to obtain acceptable fitting parameters. We thus had to use a binning factor of 4 to 10 pixels, corresponding to regions ranging from 0.8×0.8 µm to 1.9×1.9 µm. While these dimensions could in some cases extend beyond those of a spine, they were necessary to obtain sufficient photons for fitting with a single exponential function. Since the number of photons in the pixels located in synaptic regions typically ranged from 1000 and 4000 photons/pixel, a reliable multi-exponential analysis was not possible [25]; however we expected this range of photons to be sufficient for a single exponential analysis [25].

We thus set a minimum threshold of 1000 photon per pixel (corresponding to ∼10 photons at the peak, as set in the SPCImage software) in order to minimize lifetime calculation errors and to reject background signal coming from untransfected neurons. The same measured instrumental response function was used for each set of experiment. Each FLIM image was then exported as a matrix of lifetimes and photon counts. We developed a custom program in MatLab (Mathworks) to blindly detect and analyze synaptic regions. First, the PSD95-mCherry confocal image was analyzed and segmented (regions larger than 5 µm^2^ and smaller than 0.02 µm^2^ were rejected). This segmented image was then used as a mask to obtain the lifetime distribution in each synaptic ROI. The median of this distribution was scored for each synapse, and all medians of the detected synapses, 40–200 per neuron, were averaged. For living neurons, only one image per neuron was taken at different time points (10–50 detected synapses). To minimize the possible effects of photobleaching on the measured lifetimes, experiments were rejected when the intensity decreased by more than 25%. For fixed neurons, the entire dendritic tree was imaged (yielding more detectable synapses). Finally, 9–16 neurons were imaged per condition taken from 3–5 separate cultures. The histograms represent the mean (±SEM) of all neurons. In Figure S3 in file S1, representative data for the two approaches (live and fixed neurons) are plotted as bar graphs to show directly the variance obtained with our lifetime measurements and analyses.

### Spine size measurements

GluN1-GFP z-stacks were acquired just after FLIM recordings using the Zeiss LSM510 confocal microscope (excitation at 488 nm, 0.5 µm interval, optical slice of 1 µm, 8–20 planes). Analysis was done in MetaMorph (Molecular Devices, Downingtown, PA). Each image of the recorded z-stacks was first deconvolved and a maximum projection was obtained. Spine contours were traced manually, blind of the conditions, using an inclusive threshold to better delineate spines (the same value was used for each neuron). Values presented in histograms are the average of all spines analyzed ± SEM.

### HEK293 cell transfection and imaging

**Figure 2:**
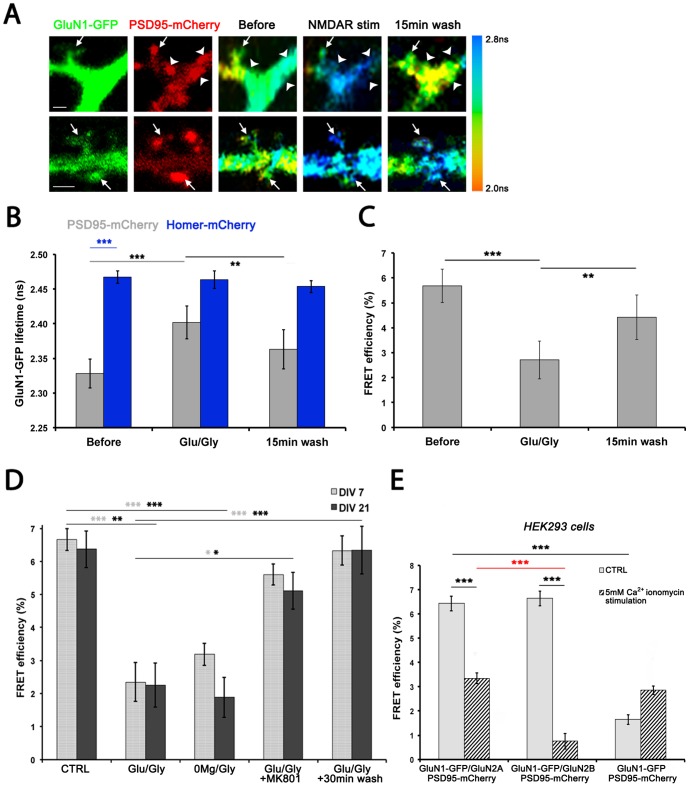
The interaction between PSD95 and the NMDAR is transiently disrupted upon NMDAR stimulation. (A) Two examples of confocal images of 14 DIV dendrites expressing GluN1-GFP (first column) and PSD95-mCherry (second column). Next are shown corresponding FLIM images of the same dendrites before (third column), just after stimulation (fourth column) and 15 min after washing out the stimulation solution (last column). The arrows point to spines in which the lifetime increased upon stimulation, the arrowheads point to dendritic shaft synapses in which the lifetime is also increased by stimulation. Color-coding represents GluN1-GFP lifetime from 2 ns to 2.8 ns (columns 3 and 4). Scale bar is 1 µm. (B) GluN1-GFP lifetime in PSD95-mCherry clusters increases upon 1–2 min Glu/Gly stimulation and decreases after 15 min wash (gray, 16 live neurons). Repeated measures ANOVA was performed (p<0.0001), followed by Bonferroni post hoc test. ** indicates p <0.01 and ***  =  p<0.001 through the figure. GluN1-GFP lifetime is significantly higher in Homer-mCherry clusters than in PSD95-mCherry clusters (dark blue, 15 live neurons). One-way ANOVA test with 6 groups was performed (p<0.0001), followed by Bonferroni post hoc test. Before stimulation and 15 min after, PSD95-mCherry neurons are statistically different from Homer-mCherry neurons (p<0.001 and p<0.01 respectively). Glu/Gly stimulation and washing had no effect on GluN1-GFP lifetime in Homer-mCherry clusters (Repeated measures ANOVA test was not significant). (C) FRET efficiency in GluN1-GFP/PSD95-mCherry expressing cells (same neurons as in B, calculated with GluN1-GFP/Homer-mCherry as the donor lifetime (experiments done the same day)) decreases upon 1–2 min Glu/Gly stimulation and increases again after 15 min wash. Repeated measures ANOVA was performed (p<0.0001), followed by Bonferroni post hoc test. (D) In DIV7 (light gray) and DIV21 (dark gray) neurons, FRET efficiency decreases after 1–2 min Glu/Gly stimulation and 5 min 0Mg^2+^/Gly stimulation. Neurons were fixed and mounted prior to imaging (see Methods). After 30 min of wash in High Mg^2+^ solution, FRET is back to basal levels. MK-801 blocks the FRET loss. Each bar in the histogram is the mean FRET efficiency of at least 10 neurons (one mean value/neuron obtained from ≈40–200 synapses), taken from at least 3 separate animal preparations. Kruskal-Wallis test was performed on both DIV7 and DIV21 groups p<0.0001 for both ages. Dunn's test was used for post hoc comparison. (E) The interaction between the NMDAR and PSD95 is mediated via an interaction with GluN2A or GluN2B and is dissociated by Ca^2+^ influx in HEK293 cells. The cells were co-transfected with the indicated constructs and treated as described in the methods. To confirm the differences between groups, Kruskal-Wallis test was performed (p<0.0001), followed by Dunn's post hoc test.

Human embryonic kidney 293 (HEK293) cells were grown to 30–40% on poly-D-lysine coated Aclar coverslips (13 mm in diameter) in DMEM growth media supplemented with 10% fetal bovine serum and Glutamax-1 (Invitrogen). Cells were transfected using Lipofectamine 2000 as described by the manufacturer and treated 16–24 h later. For Figure 2E, cells were incubated in a solution containing HBSS supplemented with 5 mM CaCl_2_ and 10 µM ionomycin (1∶1000 dilution of a methanol stock) or a solution containing only HBSS for 5 min. Cells were then immediately fixed for 10 min in methanol (−20C), washed in PBS and mounted with Prolong Gold. For FLIM analysis, all detected photons for each cell were summed into a single histogram from which one mean lifetime was obtained.

### Statistics

Data sets were first tested for normality using the Lilliefors test in Matlab (Mathworks). In case of normal distribution, a two-tailed Student T-Test was used (Excel). If more than two data sets needed to be compared, a one-way ANOVA was performed and Bonferroni's test was used for post hoc comparison (GraphPad Prism 4.0 software was used for these analyses). For non-normal distributed data sets, a Wilcoxon rank sum test was done using Matlab. For multiple comparisons, a Kruskal-Wallis test was performed followed by Dunn's post hoc test (GraphPad Prism).

## Results

### Probes for measuring interaction between NMDAR and PSD95 in spines using FRET-FLIM

To study the interaction between the NMDAR and PSD95 with FRET-FLIM, we tagged the c-terminus of GluN1 with monomeric GFP (mGFP), as a FRET donor, and tagged the c-terminus of PSD95 with mCherry as a FRET acceptor. Although PSD95 was shown to interact with the NMDAR via the GluN2 subunits [26], we did not tag their c-termini because the GFP at this position interfered with expression and trafficking of the GluN2 subunits [15], [26]. Since GluN1 forms a receptor complex with GluN2, we reasoned that by being at the c-terminus of GluN1, GFP might be close enough to PSD95-mCherry, once bound to a GluN2 subunit, to produce FRET. We thus compared spines of neurons expressing either GluN1-GFP alone or co-expressing either mCherry, PSD95-mCherry, or Homer-mCherry. As shown in Figure 1A, shorter lifetimes, indicated as orange-red, are seen only in GluN1-GFP/PSD95-mCherry expressing spines. To quantify this, the fluorescence lifetime of each pixel within one spine (as in Figure 1B) was pooled into a weighted distribution histogram (Figure 1C); the median of the distribution was then averaged for several spines from each condition (Figure 1D). The results show that GluN1-GFP lifetime is significantly shorter when PSD95-mCherry is co-expressed. Since FRET between GluN1-GFP and PSD95-mCherry was greatly increased by GluN2 subunits co-expression in HEK293 cells (Figure 2E, Figure S1B in File S1), these results suggest that native GluN2 subunits in neurons assembled with GluN1-GFP to mediate the interaction with PSD95-mCherry. However, because proteins tightly packed together in the PSD, one might think that any pair of labeled PSD proteins would show FRET to a certain extent. To verify this, we chose to label Homer with mCherry because its location, determined by light microscopy, in spines is almost indistinguishable from PSD95-mCherry (See Fig. S4 in file S1). Shiraishi et al also showed that dendritic and synaptic clustering of Homer was similar to GluN2B and PSD95 [27]. By contrast, the co-expression of Homer-mCherry (and mCherry alone) showed no measurable effect on GluN1-GFP lifetime (Figure 1D).

We can calculate the FRET efficiency (EFRET) for each condition using the following formula: EFRET =  1- (τ_DA_/τ_D_) (τ_DA_ is the lifetime of the FRET donor, GluN1-GFP, in the presence of a FRET acceptor; τ_D_ is the lifetime of GluN1-GFP expressed alone). For GluN1-GFP/mCherry and GluN1-GFP/Homer-mCherry controls, the FRET efficiency was not statistically different from GluN1-GFP alone (0%). In contrast, the FRET efficiency for GluN1-GFP/PSD95-mCherry was nearly 20 times higher compared to those control conditions (Figure 1E). This strong positive difference in FRET, when comparing GluN1-GFP/Homer-mCherry with GluN1-GFP/PSD95-mCherry is consistent with an interaction between PSD95 and the NMDAR, as reported earlier [3], [15], [16].

### FRET between GluN1-GFP and PSD95-mCherry is transiently reduced upon NMDAR stimulation

NMDAR activation was shown to transiently drive a fraction of PSD95 out of spines [5]. To determine whether the interaction between the NMDAR and PSD95 is altered by activation of the receptor, we stimulated NMDARs in neurons. Figure 2A shows GluN1-GFP and PSD95-mCherry expression in two dendrites, along with fluorescent lifetime images illustrating GluN1-GFP lifetime in the same dendrites before, immediately after a 1 min glutamate/glycine (Glu/Gly) stimulation and after a 15 min wash. To make the analysis faster and unbiased, PSD95-mCherry puncta were detected using a custom software-based method and the GluN1-GFP lifetime was evaluated in each puncta (see Methods for details). Figure 2A–C shows that GluN1-GFP lifetime increased and FRET efficiency decreased upon Glu/Gly stimulation, suggesting that the amount of interaction between GluN1-GFP and PSD95-mCherry decreased in synapses. After 15 minutes of washing with an external solution that blocks most NMDAR-mediated synaptic Ca^2+^ influx (5 mM Mg^2+^/0.6 mM Ca^2+^), GluN1-GFP lifetime decreased, suggesting a partial recovery of the interaction between PSD95 and the NMDAR. By contrast, applying the same Glu/Gly stimulation protocol to GluN1-GFP/Homer-mCherry expressing neurons had no effect on GluN1-GFP lifetime (Figure 2B).

Incubation of the neurons with MK801 (NMDAR open-channel blocker) indicated that the Glu/Gly-evoked disruption of the NMDAR/PSD95 interaction was dependent on NMDAR-gated Ca^2+^ influx (Figure 2D). To test whether targeting only synaptic NMDARs could equally disrupt the interaction, we treated the cultures with external solution without Mg^2+^ and with glycine (0Mg^2+^/Gly) [22], [28]–[30]. The results indicated that 0Mg^2+^/Gly also produced a reduction in FRET efficiency (Figure 2D). Finally, an extension of the wash period to 30 min after the initial stimulation resulted in a FRET efficiency that recovered to basal levels, demonstrating the transient characteristic of the process.

One of the hallmark features of the NMDAR in developing postnatal hippocampal and cortical neurons is the increasing ratio of GluN2A/GluN2B [10]–[13]. This developmental switch was also seen in our cultures; we measured a two-fold increase in the GluN2A/GluN2B ratio from DIV7 to DIV21 (Figure S5 in file S1). This raises the question of whether such change in ratio would affect the NMDAR activity-dependent disruption of the NMDAR-PSD95 interaction. We thus compared two developmental stages, DIV7 and DIV21, and found no significant difference in the stimulus-induced changes in FRET efficiencies between the two ages (Figure 2D). It is probable that PSD95 can interact with the NMDAR via both GluN2 subunits [5], [15], [16], [31]. We tested whether Ca^2+^ influx in HEK cells can disrupt the NMDAR-PSD95 interaction through either GluN2 subunit. The results indicated that i) both GluN2A and GluN2B could support FRET between GluN1-GFP and PSD95-mCherry and that ii) this interaction was disrupted by Ca^2+^ influx (Figure 2E). Taken together, these results show that NMDAR/PSD95 interaction is transiently disrupted upon NMDAR stimulation both in young and mature hippocampal neurons, a process that likely reflects dynamic interactions via both GluN2A and 2B. It should be noted that the measured decrease in FRET between PSD95 and the NMDAR could also involve a conformational change.

### CaMKII regulates the NMDAR/PSD95 interaction by two distinct mechanisms during synaptic development

**Figure 3:**
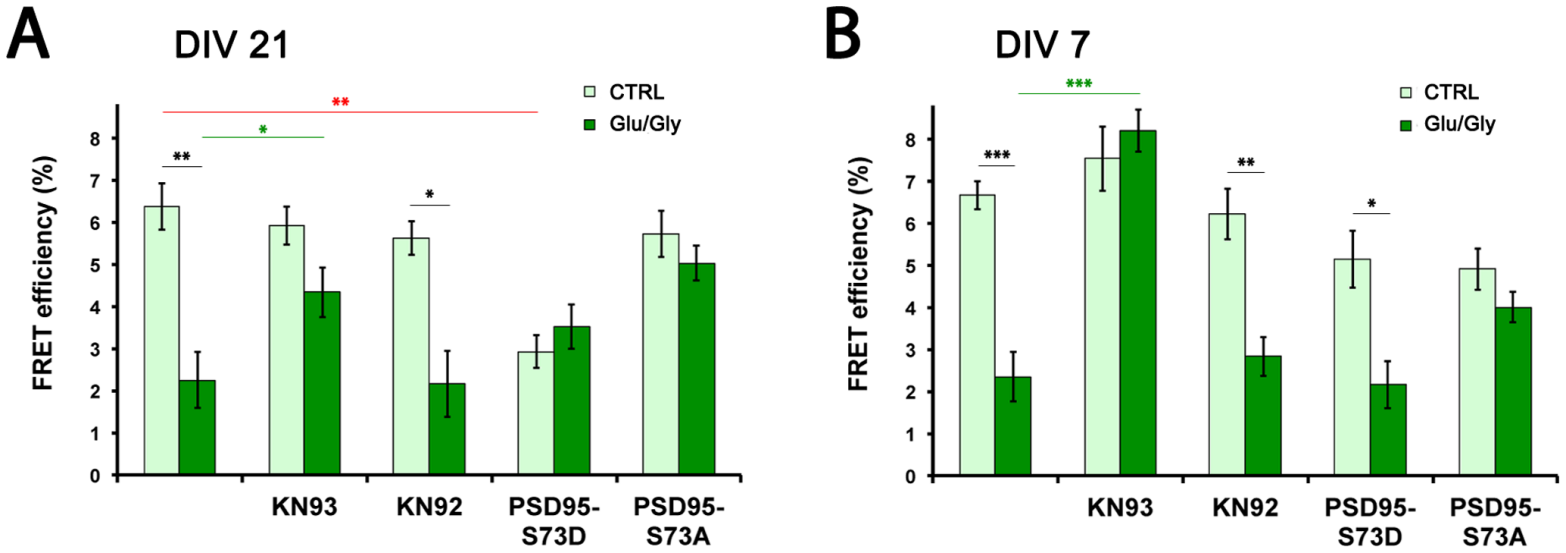
CaMKII regulates the NMDAR/PSD95 interaction by distinct mechanisms during synaptic development. (A) CaMKII inhibition with KN93 (10 µM) and PSD95 phosphorylation reduce the activity-dependent dissociation of PSD95 from the NMDAR in DIV21 neurons. The inactive drug KN92 (10 µM) gives results similar to control. NMDAR interaction with PSD95-S73D is much less than with PSD95-WT. In contrast, PSD95-S73A interacts with the NMDAR and FRET does not change upon stimulation. Light green, control unstimulated; dark green, 1–2 min Glu/Gly stimulation. Statistical analysis by Kruskal-Wallis test (p<0.0001), followed by Dunn's post hoc test * indicates p<0.05, ** p<0.01 and *** p<0.001. (N = 10–14 neurons per condition). (B) In DIV7 neurons, CaMKII inhibition also reduces the activity-dependent dissociation of PSD95 from the NMDAR, whereas PSD95-S73D interacts with the NMDAR as well as PSD95-WT does (compare unstimulated CTRL vs PSD95-S73D, p>0.05), the 1–2 min Glu/Gly stimuli disrupting the interaction. PSD95-S73A mutant does not dissociate from the NMDAR upon stimulation. Statistical analysis by one-way ANOVA test (p<0.0001), followed by Bonferroni post hoc test * indicates p<0.05, ** p<0.01 and *** p<0.001. (N = 10–22 neurons per condition).

We next sought to understand the mechanisms by which the interaction between the two proteins is regulated upon NMDAR stimulation. Since CaMKII is known to play a role in regulating PSD95 trafficking [5], we used KN93 to test whether CaMKII influences NMDAR/PSD95 interaction. KN93, but not the inactive analogue KN92, impaired the FRET loss normally seen upon stimulation, both in DIV21 and DIV7 neurons, suggesting that CaMKII activation is needed to disrupt the interaction between NMDAR and PSD95 (Figure 3A and B). Interestingly however, the effect of the competitive inhibitor KN93 seemed less pronounced in DIV21 neurons, which could be due to the important increase in CaMKII expression between DIV7 and DIV21 [32].

Gardoni and colleagues showed that CaMKII-dependent phosphorylation of PSD95 at S73 affects the NMDAR/PSD95 interaction [16]. The authors observed that the mutant mimicking a permanently phosphorylated PSD95 (S73D) colocalized much less with GluN2A compared to PSD95-WT in HEK cells, whereas its colocalization with GluN2B was undistinguishable from PSD95-WT [16]. Moreover, Steiner et al observed that the PDS95-S73A mutant, mimicking a non-phosphorylable form, was stable in the spine and did not leave upon stimulation, whereas the S73D mutant was trafficked out of the spine much faster than PSD95-WT in basal conditions, stimulation not affecting the transfer rate [5]. We thus tested PSD95-S73A/D mutants tagged with mCherry in our FRET-FLIM assay. In mature neurons, PSD95-S73D mutant had little basal interaction with the NMDAR, and no change was seen upon stimulation (Figure 3A). However, PSD95-S73A did interact with the receptor in unstimulated neurons and stimulation did not disrupt the interaction (Figure 3A). This suggests that CaMKII phosphorylation of PSD95 at S73 regulates the NMDAR/PSD95 interaction in DIV21 cultured neurons.

In contrast, in young neurons (DIV7), CaMKII-dependent phosphorylation of PSD95-S73 did not seem to regulate NMDAR/PSD95 interaction in the same manner. Indeed, both PSD95-S73D and S73A mutants interacted with the NMDAR to a similar extend as PSD95-WT in basal conditions, and a disruption of the interaction was caused by 1–2 min Glu/Gly stimulation for the S73D, but not for S73A (Figure 3B). One possible interpretation of these results is that the changing ratio of GluN2 subunits during development alters the mechanisms of PSD95-NMDAR interaction. Indeed, phosphorylation of PSD95-S73 is unlikely to impact on it binding to GluN2B [16], which is the dominantly expressed GluN2 subunit at DIV7. Overall, these results suggest that while CaMKII can impact on NMDAR-PSD95 interaction, other NMDAR-dependent signaling processes are likely involved, since for instance, the pre-phosphorylated PSD95 (S73D) still dissociates during NMDAR stimulation.

### Calpain and Src family kinases control the NMDAR/PSD95 interaction

**Figure 4:**
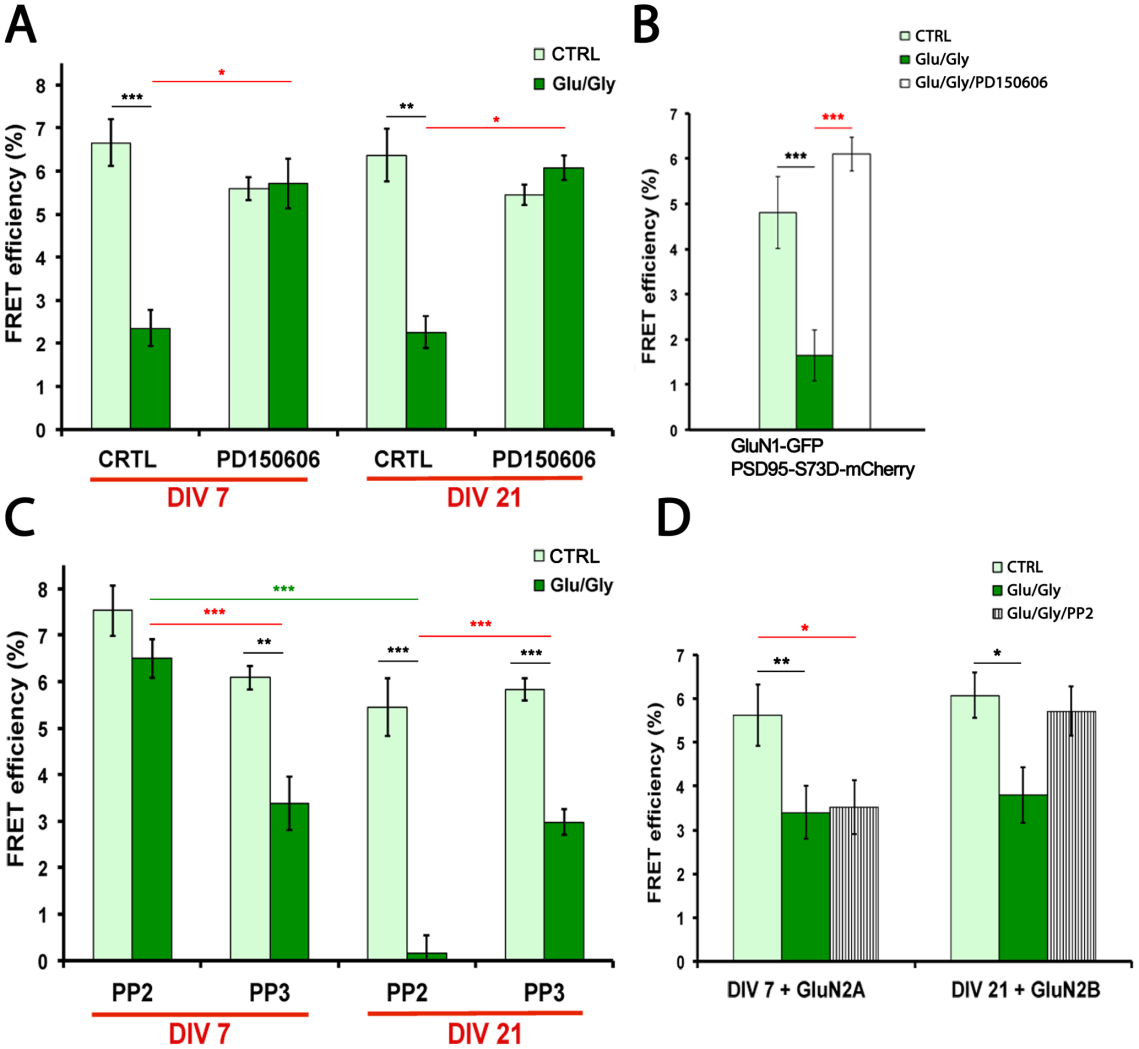
Calpain mediates the activity-dependent dissociation of PSD95 from the NMDAR and Src family kinases differentially control this process during development. (A) Calpain inhibition with PD150606 (10 µM) prevents the activity-dependent dissociation of PSD95 from the NMDAR both in DIV7 and DIV21 neurons. Light green, control unstimulated; dark green, stimulated for 1–2 min with Glu/Gly. A Kruskal-Wallis test revealed significant differences in the data set (p<0.0001). Dunn's post hoc test showed differences induced by Glu/Gly stimulation and calpain inhibition. * indicates p<0.05, ** p<0.01 and ***p<0.001 through the figure. (N = 10–14 neurons per condition). B) Calpain inhibition also prevents the activity-dependent dissociation of PSD95 from the NMDAR in DIV7 neurons expressing PSD95-S73D-mCherry. One-way ANOVA test was performed, revealing differences in the data sets (p<0.0001). Bonferroni was used as a post hoc test. *** indicates p<0.001. (N = 9–11 neurons per condition). (C) Src family kinase inhibition with PP2 (10 µM) prevents the activity-dependent dissociation of PSD95 from the NMDAR in DIV7 neurons, whereas it increases it in DIV21 neurons. The inactive analog PP3 (10 µM) does not influence the dissociation. One-way ANOVA was performed (p<0.0001) followed by Bonferroni post hoc test. (N = 10–14 neurons per condition). (D) PP2 no longer has an effect in DIV7 neurons overexpressing GluN2A, while it now blocks dissociation in DIV21 neurons overexpressing GluN2B. One-way ANOVA revealed significant differences between the groups (p<0.001), Bonferroni test was used for post hoc comparisons. (N = 10–11 neurons per condition).

What NMDAR activity-dependent signaling process, other than CaMKII activation, could also disrupt the NMDAR-PSD95 interaction? It was previously demonstrated that NMDAR stimulation can cause cleavage of the GluN2 c-tails by calpain in cultured hippocampal neurons [8], [9]. To investigate whether calpain regulates the NMDAR-PSD95 interaction in spines, we incubated the neurons with calpain inhibitor PD150606. Figure 4A shows that this treatment completely blocked the activity-dependent dissociation of the NMDAR-PSD95 complex, both in DIV7 and DIV21 cultures. Another organic calpain inhibitor (MDL-28170) also blocked this dissociation (in DIV7 neurons incubated with 50 µM MDL, FRET efficiency was 6.3±0.7 without stimulation (N = 10 neurons) and 7.6±0.7 with 1–2 min Glu/Gly (N = 9 neurons); p = 0.21, unpaired t-test), validating further the specificity of this calpain inhibition. In addition, over-expression of the natural inhibitor calpastatin largely reduced this dissociation (in DIV7 neurons expressing only NR1-GFP and PSD95-mCherry, FRET efficiency dropped by ∼3 fold with Glu/Gly, Fig 4A, whereas in calpastatin-transfected neurons, FRET efficiency dropped only by ∼1.4 fold: 8.1±0.9 without stimulation (N = 10 neurons) vs 5.8±0.7 with 1–2 min Glu/Gly, (N = 10 neurons)). Thus, calpain activity can be another mechanism by which the NMDAR/PSD95 interaction is disrupted, even in mature neurons. It is noteworthy that KN93 was shown not to inhibit calpain activity in cultured neurons [33], suggesting that CaMKII is not acting directly on calpain activity. Furthermore, while we found that phosphorylation of PSD95, as mimicked by the mutation S73D, does not impact on NMDAR-PSD95 interaction in young neurons, the mutant can still dissociate upon NMDAR activation (Fig 3A) and this dissociation is also calpain-dependent (Fig 4B). Since calpain appears to preferentially cleave GluN2 subunits over PSD95 [34], we might expect the receptor to be the first target for cleavage.

If indeed calpain cleavage of GluN2 subunits regulates the NMDAR-PSD95 interaction, we would therefore expect that the Src family kinase (SFK) would also regulate the interaction, since SFK has been shown to differentially regulate calpain cleavage of the two GluN2 subunits [7], [35]. Tyrosine phosphorylation of GluN2B c-terminal domain is thought to enhance its calpain cleavage, whereas that of GluN2A is thought to reduce its cleavage [7]. To test the involvement of SFK in the NMDAR activity-dependent regulation of NMDAR-PSD95 interactions in spines, we incubated the neurons with SFK inhibitor PP2, or its inactive analog PP3. In young neurons, SFK inhibition protected the NMDAR/PSD95 interaction in spines following NMDAR stimulation (Figure 4C). In contrast, PP2 further increased the complex dissociation in spines upon stimulation in DIV21 neurons (Figure 4C). Given that the ratio of GluN2A/GluN2B increases during development, these results are consistent with previous indication that calpain cleavage of GluN2B in young neurons is promoted by SFK activity [35], whereas cleavage of GluN2A in mature neurons is reduced by SFK activity [7]. This interpretation is however circumstantial. We reasoned that over-expressing GluN2A in DIV7 cultures or GluN2B in DIV21 cultures (along with GluN1-GFP and PSD95-mCherry) might allow to further validate the GluN2 differential SFK regulation hypothesis. When GluN2A was over-expressed in DIV7 cultures, PP2 did not block the NMDAR activity-dependent dissociation of the NMDAR/PSD95 interaction in spines. Meanwhile, in DIV21 cultures, GluN2B over-expression completely reversed PP2 effect (Figure 4D). These results support the interpretations that i) GluN2 subunit cleavage by calpain is involved in the activity-dependent dissociation of NMDAR/PSD95 complex, and that ii) the GluN2 subunit turnover between DIV7 and DIV21 is responsible for the differential role of SFK on NMDAR/PSD95 interaction.

### Calpain-dependent separation of NMDAR-PSD95 correlates with spine volume increase

**Figure 5:**
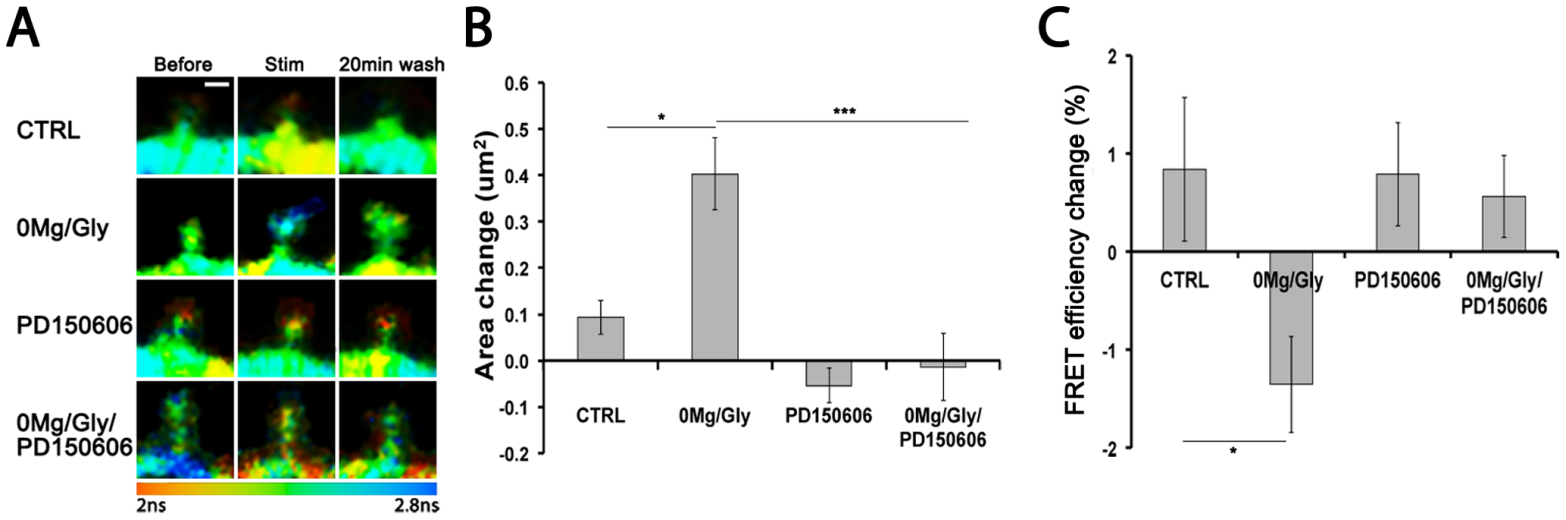
Calpain is essential for activity-dependent spine remodeling. (A) FLIM images of control spines (top), spines from neurons stimulated with 0Mg^2+^/Gly for 5 min (second row), spines from neurons treated with PD150606 (third row), and spines from neurons treated with PD150606 and stimulated with 0Mg^2+^/Gly for 5 min (last row). Scale bar is 1 µm. Color coding represents GluN1-GFP lifetime from 2 ns to 2.8 ns. (B) Spine area change (area 20 min after stimulation – area before) in control spines kept in blocking solution for the same time (112 spines/14 neurons), 0Mg^2+^/Gly stimulated spines (99s/15N), PD150606 treated spines (132 s/14 N) and PD150606 treated and stimulated with 0Mg^2+^/Gly spines (126 s/14 N). Statistical analysis performed by Kruskal-Wallis (p<0.0001) followed by Dunn's test. *indicates p<0.05 and *** =  p<0.001. (C) FRET efficiency change after the 0Mg^2+^/Gly LTP stimulation (%FRET efficiency after – %FRET efficiency before) in the same neurons as in B. One-way ANOVA (p<0.02) followed by Bonferroni post hoc test, p<0.05 between CTRL and 0Mg^2+^/Gly.

Since the phosphorylation of PSD95 has been shown to control long-lasting change in spine size and PSD95 trafficking in organotypic slices [5], we wondered whether calpain activity during synaptic activity could play a role in spine plasticity. We showed previously that synaptic NMDAR stimulation and CaMKII activation can induce long-lasting change in spine volume in cultured hippocampal neurons [32]. We thus measured both the area projected from GluN1-GFP expressing spines (see Methods) and FRET change between GluN1-GFP and PSD95-mCherry in the same neurons. Figure 5B shows that while basal activity produced little change in spine size, 5 min of enhanced synaptic NMDAR activity (0Mg^2+^/Gly) led to a 0.40±0.08 µm^2^ increase in size 20 min later, an effect that was blocked by inhibiting calpain with PD150606. In the same spines, the FRET efficiency between GluN1-GFP and PSD95-mCherry decreased on average by 1.4% upon stimulation by 0Mg^2+^/Gly, but not in presence of PD150606 (Figure 5C). These results demonstrate a correlation between the synaptic NMDAR activity- and calpain-dependent disruption of NMDAR/PSD95 and long-lasting change in spine size.

## Discussion

The dynamic reorganization of the PSD during synaptic transmission is a complex process, challenging to examine in living spines and to relate to spine plasticity. In this study, we introduce the use of FRET-FLIM to assess the rules governing the dynamic changes in interactions between the NMDAR and PSD95 in dendritic spines undergoing remodeling. We show a significant level of FRET between PSD95-mCherry and GluN1-GFP inside living spines and that synaptic NMDAR activity can transiently reduce this FRET, both in young and mature synapses, as revealed by an increase in GluN1-GFP lifetime. The extent of this FRET between PSD95-mCherry from the GluN1-GFP is under the control of three families of enzymes, CaMKII, calpain and SFK, all of which have been implicated in models of long-term potentiation of synaptic transmission [2], [4], [36], [37]. During the period of post-natal synaptic maturation, the enzymatic regulation of the activity-dependent NMDAR-PSD95 separation changes in a way that is consistent with the corresponding change in GluN2A/2B ratio. The evidence presented here points toward a combined requirement for CaMKII and calpain activity for dissociating the complex. In our experiments, we cannot rule out that the activity-dependent decrease in FRET between GluN1-GFP and PSD95-mCherry is due to conformational changes rather than complete separation of PDS95 from the NMDAR. Indeed, a dynamic change in FRET can report either a change in conformation that alters the distance between the probes or a complete separation between the interacting partners. The main argument in favor of a complete separation is the involvement of calpain, which cleaves its substrates. Furthermore, a fraction of PSD95 was shown to transiently exit the spines upon synaptic NMDAR activation [5]. Finally, the reversibility of the process is slow (∼30 min), more consistent with the arrival of new partners, rather than a reversing conformational change.

**Figure 6:**
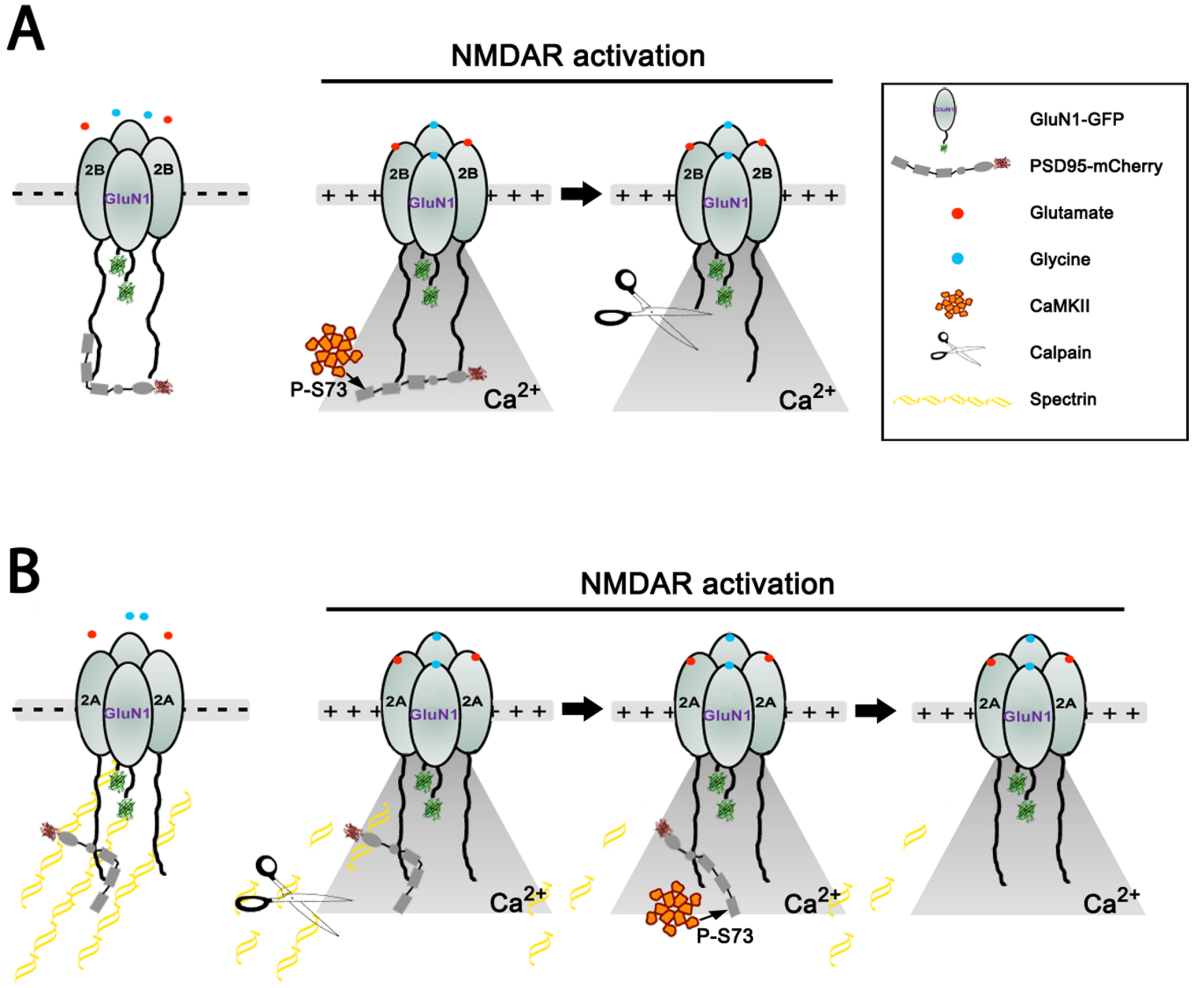
Proposed mechanism of NMDAR-PSD95 dissociation during NMDAR activation in young neurons (A) versus mature neurons (B). (A) In young neurons expressing more GluN2B than GluN2A, the PDZ2 domain of PSD95 supports its binding to GluN2B c-terminus [15]. The phosphorylation by CaMKII of S73 in the PDZ1 domain of PSD95, following NMDAR activation and Ca^2+^ influx, would repel this portion of PSD95 from the receptor, possibly where another binding site for PSD95 on GluN2B was recently identified (aa 1149–1157; [31]. This may then reduce the protective effect of PSD95 against calpain-mediated cleavage of the ctail of GluN2B [6], leading to the separation of PSD95 from the receptor. This scenario reconciles our data showing that inhibiting CaMKII (KN93) prevents the separation of PSD95 from the receptor, but that S73D-PSD95 can still bind GluN2B-containing NMDARs [16]. (B) In mature neurons, PSD95 is mainly bound to GluN2A-containing receptors, via two binding sites on the receptor: i) the c-terminal site binding to PDZ2 and ii) another site (aa 1382–1389) binding to the SH3 domain of PSD95 [31]. The phosphorylation of S73-PSD95 alone (S73D mutation) was sufficient to prevent the PSD95-NMDAR interaction in mature synapses, but CaMKII was unable to trigger the complex separation in the presence of a calpain inhibitor, suggesting that a calpain-sensitive PSD component (spectrin is a well know calpain substrate [34], [38], [39] and is thus shown merely as an example) must be removed to allow CaMKII to access S73 on PSD95.

Deciphering the relationship between the three classes of enzymes in regulating the activity-dependent separation of the PSD95-NMDAR complex may require further investigation. Our results suggest that calpain activity is necessary at both immature (DIV7) and mature (DIV21) synapses. CaMKII activity is also required at both stages. However, if the implication of CaMKII involves PSD95 phosphorylation, our results with PSD95-S73D would argue that it is not sufficient in immature synapses, but maybe sufficient in mature synapses. Meanwhile PSD95-S73A did not dissociate upon NMDAR stimulation at either developmental stage. Finally, while calpain activity appears to be required for PSD95-NMDAR dissociation, it is not sufficient, since CaMKII inhibition with KN93 prevented the process. How can these results be interpreted by a concerted CaMKII and calpain-dependent process? Clues may come from the facts that i) there is a developmental change in GluN2A/2B ratio in these neurons; ii) PSD95 was shown to protect the c-terminal domains of GluN2 against calpain in HEK cells [6], [17]; iii) Previous results suggest that GluN2B is more prone to calpain cleavage compared to GluN2A [14]; iv) calpain may cleave additional substrates at synapses, other than GluN2 (e.g. spectrin). In young synapses, dominated by GluN2B, phosphorylation of PSD95 by CaMKII should not prevent their interaction [16]. However, the phosphorylation may modify the conformation of PSD95 in a way that it would no longer protect GluN2B from calpain cleavage. Meanwhile at mature synapses, dominated by GluN2A, phosphorylation of PSD95 by CaMKII may be sufficient to prevent the complex formation [16]; the role of calpain might then be to remove proteins bound to the NMDAR complex, such as spectrin [34], [38], [39] to enable the large CaMKII enzyme to access PSD95 for phosphorylation. We present in Figure 6 a working model, based on this above interpretation, of the regulation of the NMDAR-PSD95 complex separation by calpain and CaMKII, following NMDAR activation.

CaMKII activity has also been shown to activate casein kinase II (CKII) activity, leading to GluN2B phosphorylation on Ser1480 and dissociation of PSD95, following the relief of chronic AP5 inhibition in cortical neurons [40]. We have not tested the implication of CKII in PSD95-NMDAR dissociation, which could also have a role, although glutamate stimulation was shown not to induce phosphorylation of Ser1480 [40].

Not described in Figure 6 is the impact of SFK, which we examined because of its reported differential effects on GluN2A/2B cleavage by calpain during development [7], [35]. Our findings are consistent with reported impacts of Src and Fyn on GluN2 cleavage by calpain. In young neurons, tyrosine phosphorylation via Fyn was shown to promote GluN2B cleavage [35], hence we found that SFK inhibitor PP2 helped maintain the PSD95-NMDAR interaction during NMDAR activation. In mature neurons, tyrosine phosphorylation via Src activity reduces cleavage of GluN2A [7], which is consistent with our observation that inhibiting SFK with PP2 increased even further the separation of PSD95 from the NMDAR. The over-expression of GluN2A in young neurons and that of GluN2B in mature neurons yielded further support to this opposite GluN2A/2B regulation of calpain susceptibility by SFK.

The regulation of calpain-mediated cleavage of GluN2 subunits has been largely studied by biochemical means on neurons exposed to prolonged glutamatergic stimulation [6], [8], [9], [35]. Hence, the emphasis in those studies was made on the excitotoxic forms of NMDAR cleavage, including the implication of extra-synaptic NMDARs, although the pioneers of this field suggested a putative role for calpain-mediated cleavage of NMDAR in synaptic plasticity [37], [39], [41], [42]. Our results pointing to synaptic calpain activity on GluN2 turns the emphasis of this process on its potential role in synaptic remodeling. We examined the impact of calpain on activity-dependent spine growth, using a cLTP protocol that we used previously to implicate synaptic GluN2B and CaMKII [32]. We found that calpain activity was necessary for this activity-dependent spine plasticity. Thus, CaMKII and calpain activities, which both are required for the separation of PSD95 from the NMDAR, transduce synaptic GluN2B activity into spine remodeling. These results agree with the observation that the transient exit of PSD95 from spines during synaptic NMDAR activity accompanies spine growth [5].

In the context of SFK regulation of synaptic potentiation, GluN2B cleavage could possibly be involved. It has been shown that SFK is required for the induction of LTP at CA1 synapses [4], [43], and that Fyn in particular controls metaplasticity in CA1 via regulation of GluN2B [44]. Because Fyn promotes the calpain-mediated cleavage of GluN2B [45], while we observed a reduction in PSD95-GluN2B separation in presence of SFK inhibitor PP2, we suggest that modulation of GluN2B cleavage may be part of the SFK regulation of synaptic potentiation.

The implication of calpain in LTP induction has been proposed in numerous studies but the underlying mechanisms have remained elusive and controversial [36], [41]. The cleavage of GluN2B may promote its increased mobility and exit from the synapse in cLTP [46], a process that may be essential for LTP [11]. In young neuronal circuits, LTP has been associated with spine growth, requiring NMDAR and CaMKII activities [2]. Our findings that cLTP-induced spine growth is also calpain-dependent suggest a possible convergence of these signaling processes in spine maturation. Thus, it would be worthwhile to examine if the proposed implication of calpain in LTP induction involves the dissociation between GluN2B and PSD95.

## Supporting Information

File S1 Contains the following files: Figure S1. Representative fluorescence decay curves obtained with high or low photon count demonstrate equally good fitting characteristics; the relationship between the number of photons per pixel and the calculated lifetime is different for GluN1-GFP alone vs GluN1-GFP/PSD95-mCherry. Figure S2. GluN1-GFP/PSD95-mCherry are suitable probes to detect NMDAR/PSD95 interaction both in live and fixed HEK293 cells. Figure S3. Scatter plots of representative data for live and fixed neurons. Figure S4. Expression of PSD95-mCherry and Homer-mCherry in DIV14 live hippocampal neurons. Figure S5. Synaptic GluN2A/GluN2B ratio increases during development in cultured hippocampal neurons. Supplementary methods, immunocytochemistry, related to Figure S5.
(PDF)
