# Supplementary material for: FRET-FLIM Investigation of PSD95-NMDA Receptor Interaction in Dendritic Spines; Control by Calpain, CaMKII and Src Family Kinase
Source: PLoS One. 2014 Nov 13;9(11):e112170. doi: 10.1371/journal.pone.0112170 (PMC4230936; doi:10.1371/journal.pone.0112170)
Supplement: File S1 — Contains the following files: Figure S1. Representative fluorescence decay curves obtained with high or low photon count demonstrate equally good fitting characteristics; the relationship between the number of photons per pixel and the calculated lifetime is different for GluN1-GFP alone vs GluN1-GFP/PSD95-mCherry. Figure S2. GluN1-GFP/PSD95-mCherry are suitable probes to detect NMDAR/PSD95 interaction both in live and fixed HEK293 cells. Figure S3. Scatter plots of representative data for live and fixed neurons. Figure S4. Expression of PSD95-mCherry and Homer-mCherry in DIV14 live hippocampal neurons. Figure S5. Synaptic GluN2A/GluN2B ratio increases during development in cultured hippocampal neurons. Supplementary methods, immunocytochemistry, related to Figure S5. (PDF) [file pone.0112170.s001.pdf]

SUPPORTING INFORMATION

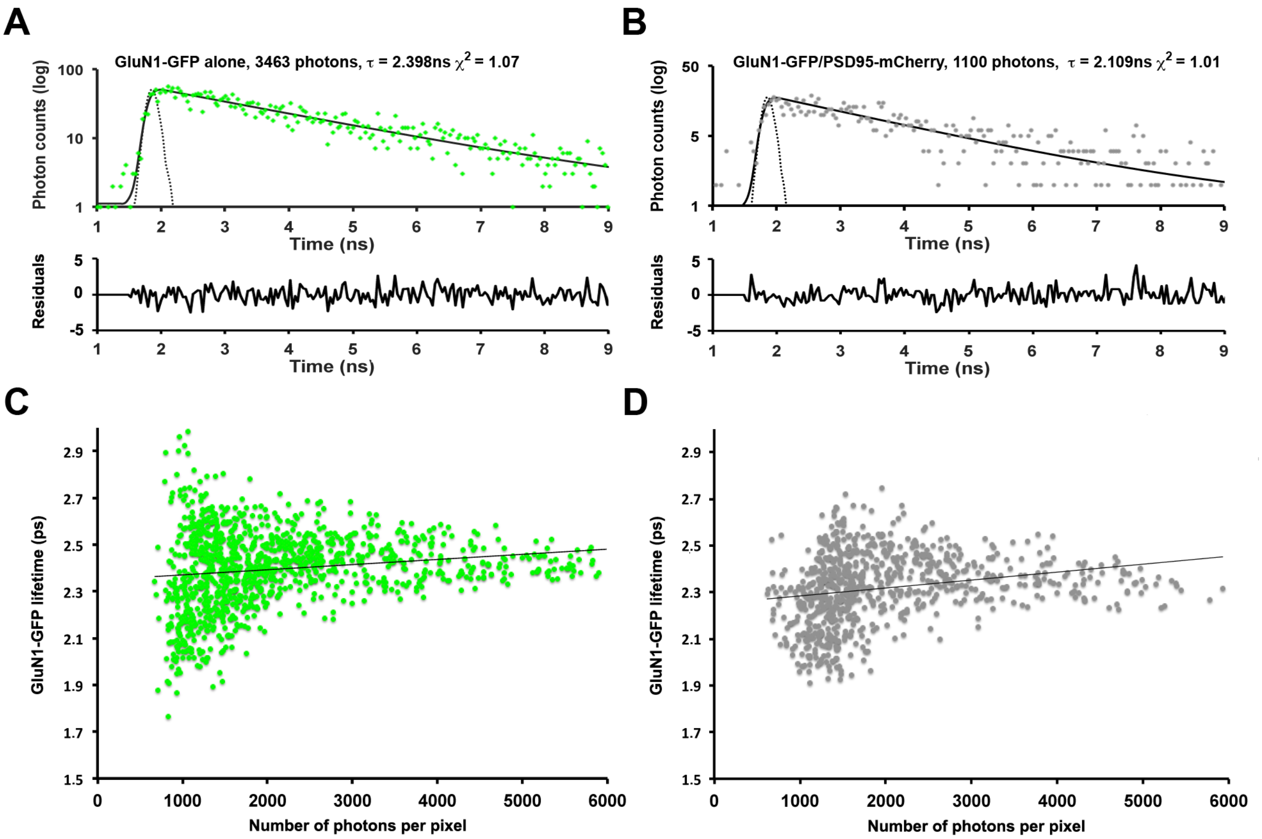

Figure S1. Representative fluorescence decay curves obtained with high or low photon count demonstrate equally good fitting characteristics; the relationship between the number of photons per pixel and the calculated lifetime is different for GluN1-GFP alone vs GluN1-GFP/PSD95-mCherry

(A) Raw data of the fitted curve shown in Fig. 1B for GluN1-GFP. The Individual detected photons are shown in green, fit curve in black. Dotted line is the instrumental response function (IRF). The residuals are shown in the bottom panel. Note the good chi-squared value and low residuals.

(B) Raw data of the fitted curve shown in Fig. 1B for GluN1-GFP/PSD95-mCherry. Detected photons are shown in gray. The IRF used is the same as in (A). Even with lower photon counts, the fitting characteristics are excellent. Note that this is a low photon count example, in GluN1-GFP/PSD95-mCherry (and in GluN1-GFP) expressing spines, the number of photons ranges from 1000-4000 photons per pixel.

(C) Graph of the calculated lifetime vs the number of photons per pixel in one image of a GluN1-GFP expressing neuron containing a total of 21 spines and 1148 pixels in these selected spines. The calculated lifetime varies very slightly with the number of photons used in each fit or pixel. The mean lifetime for a pixel of 1000 photons is 2.37ns and 2.44 for a pixel of 4000 photons.

(D) As in (C) but using a GluN1-GFP/PSD95-mCherry expressing neuron image containing 14 spines and 744 pixels. As one could expect, FRET changes the data distribution and the slope of the linear regression. Brighter pixels are

more likely to not contain acceptor proteins and thus should show less FRET. This results in a mean lifetime of 2.28ns for pixels with an intensity of 1000 photons and 2.39ns for 4000 photons.

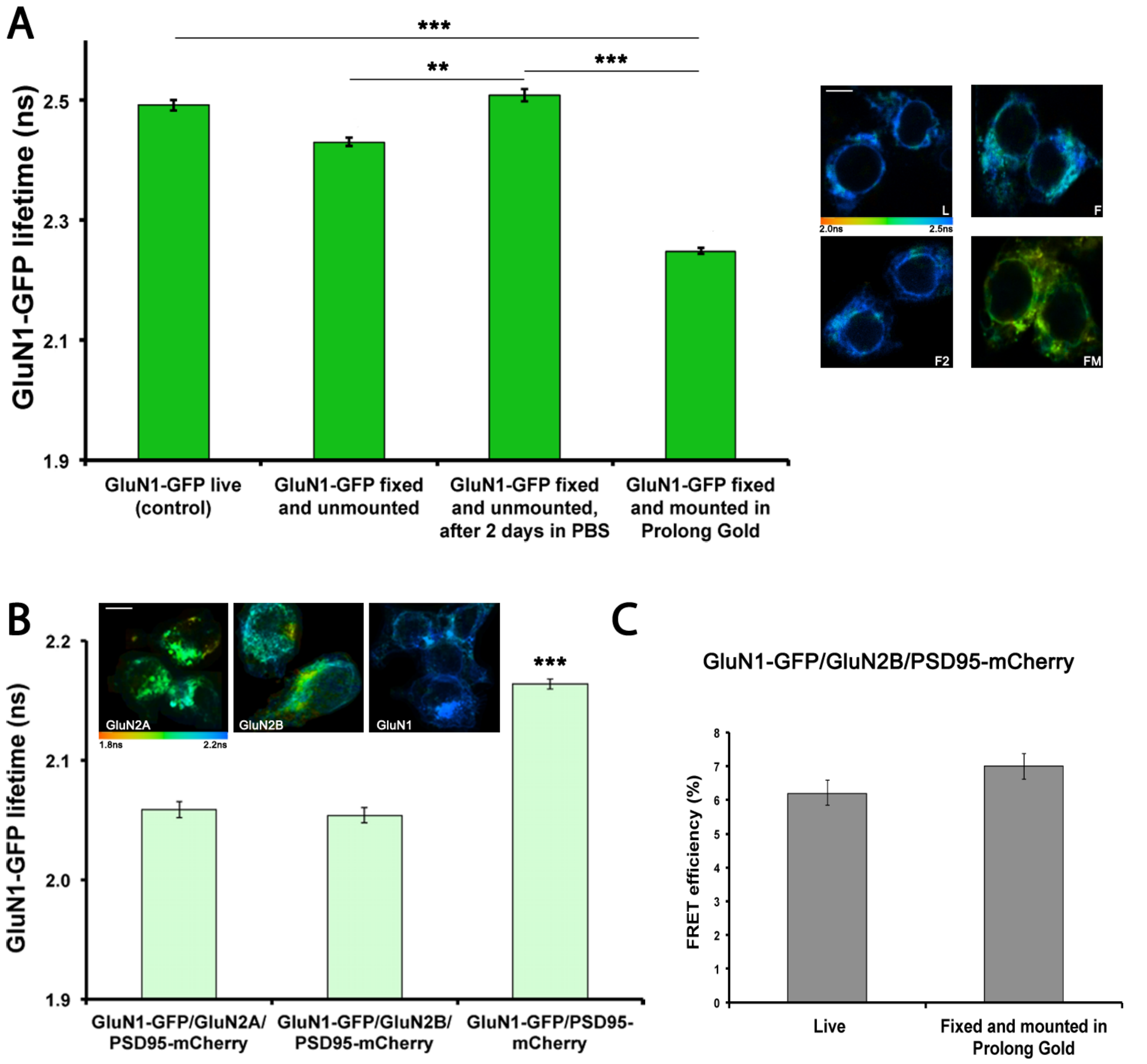

Figure S2. GluN1-GFP/PSD95-mCherry are suitable probes to detect NMDAR/PSD95 interaction both in live and fixed HEK293 cells

(A) Lifetime of GluN1-GFP in HEK293 cells. Fixation alone reduces GluN1-GFP lifetime, this effect is reversed after two days in PBS; Prolong Gold high refractive index further reduces GluN1-GFP lifetime. On the right are displayed representative FLIM images of GluN1-GFP lifetime in live cells (L, top left), Fixed and unmounted (F, top right), Fixed

after 2 days in PBS (F2, bottom left), Fixed and mounted in Prolong Gold (FM, bottom right). Scale bar is 15μm. Color coding represent GluN1-GFP lifetime from 2.0ns (red) to 2.5ns (blue). Statistical analysis was made by Kruskal-Wallis ($p<0.0001$), followed by Dunn's *post hoc* test. *** indicates $p<0.001$ and ** $p<0.01$.

(B) Lifetime of GluN1-GFP in HEK293 cells (fixed and mounted in Prolong Gold) expressing PSD95-mCherry is shorter when GluN2A or GluN2B are co-expressed, suggesting that NMDAR interacts with PSD95 via both GluN2A and GluN2B but not via GluN1. Kruskal-Wallis test was performed ($p<0.0001$), followed by Dunn's *post hoc* test. Inset displays representative FLIM images of GluN1-GFP lifetime in fixed cells co-expressing GluN2A and PSD95-mCherry (GluN2A, first image); GluN2B and PSD95-mCherry (GluN2B, second image); PSD95-mCherry (GluN1, last image). Scale bar is 10μm. Color coding represent GluN1-GFP lifetime from 1.8ns (red) to 2.2ns (blue).

(C) FRET efficiency in HEK293 cells co-expressing GluN1GFP/GluN2B/PSD95-mCherry; calculated with EFRET= (1-(τ_{DA}/τ_D)*100 (τ_{DA} is the lifetime of the FRET donor, GluN1-GFP in the presence of PSD95-mCherry and GluN2B in each cell; τ_D is the lifetime of the FRET donor, GluN1-GFP, when expressed alone, averaged in all cells (either live cells or fixed and mounted in Prolong Gold cells from experiment shown in A)). FRET efficiency is similar in both conditions; unpaired two-tailed Student Ttest was not significant ($p=0.46$).

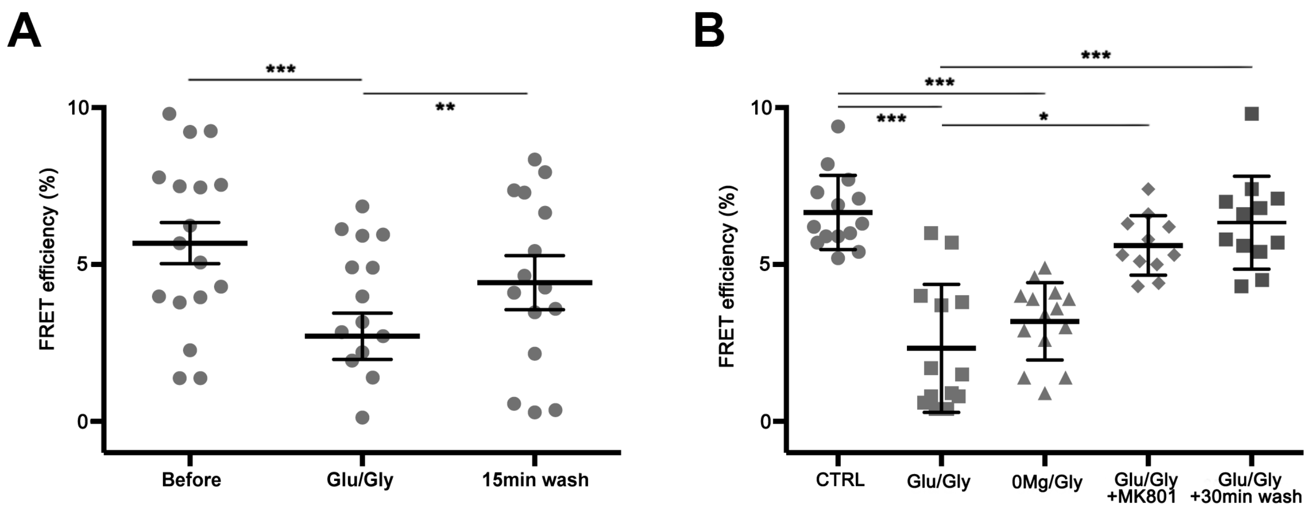

Figure S3. Scatter plots of representative data for live and fixed neurons

(A) Data shown in Fig. 2C represented as a scatter plot. Live neurons expressing GluN1-GFP and PSD95-mCherry were imaged before, during and after Glu/Gly stimulation. FRET efficiency in GluN1-GFP/PSD95-mCherry expressing neurons decreases upon 1-2min Glu/Gly stimulation and increases again after 15min wash. Each data point is the mean FRET efficiency of each neuron (one mean value/neuron obtained from ≈10-50 synapses, see methods), taken from 5 separate animal preparations. Repeated measures ANOVA was performed ($p<0.0001$), followed by Bonferroni *post hoc* test.

(B) Data shown in Fig. 2D for DIV7 neurons represented as a scatter plot. FRET efficiency decreases after 1-2min Glu/Gly stimulation and 5min $0Mg^{2+}$/Gly stimulation. Neurons were fixed and mounted prior to imaging (see methods). After 30 min of wash in High Mg^{2+} solution, FRET is back to basal levels. MK-801 blocks the FRET loss. Each data point is the mean FRET efficiency of each neuron (one mean value/neuron obtained from ≈40-200 synapses), taken from at least 3 separate animal preparations. Kruskal-Wallis test was performed ($p<0.0001$), followed by Dunn's *post hoc* test.

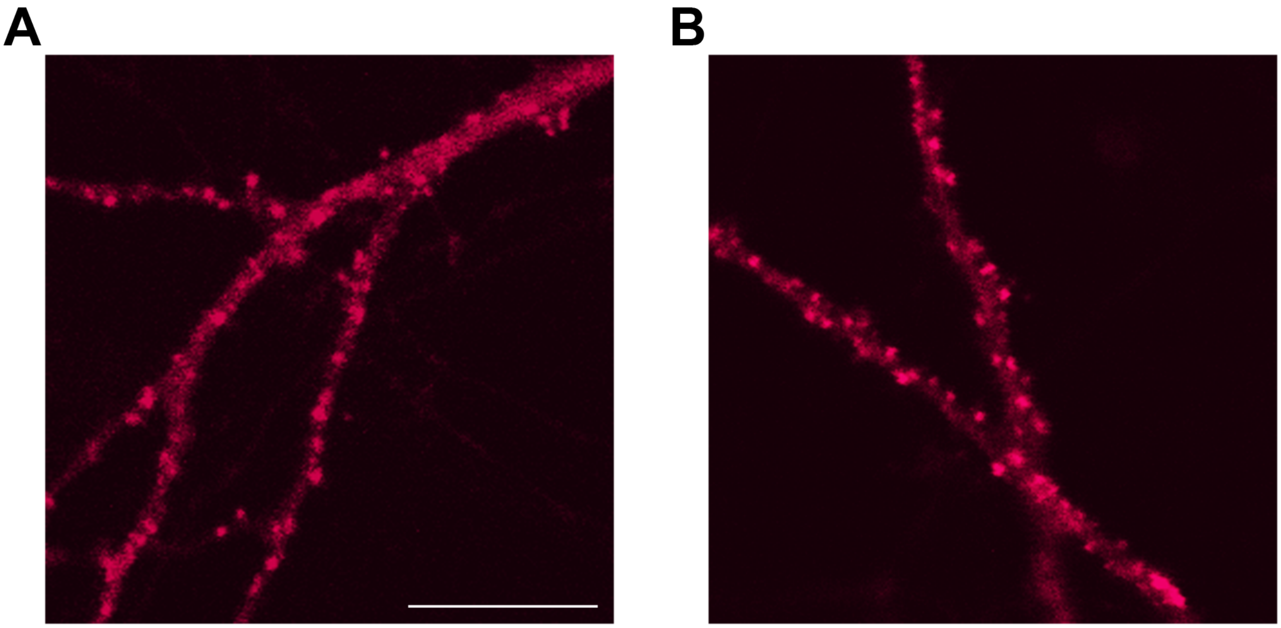

Figure S4. Expression of PSD95-mCherry and Homer-mCherry in DIV14 live hippocampal neurons

(A) Confocal image of a live DIV14 hippocampal neuron expressing PSD95-mCherry. Synaptic PSD95 clusters are seen both in spines and dendrites and are easily distinguishable from cytoplasmic diffuse expression. Scale bar is 10µm.

(B) Live DIV14 hippocampal neuron expressing Homer-mCherry imaged with the same settings as in (A).

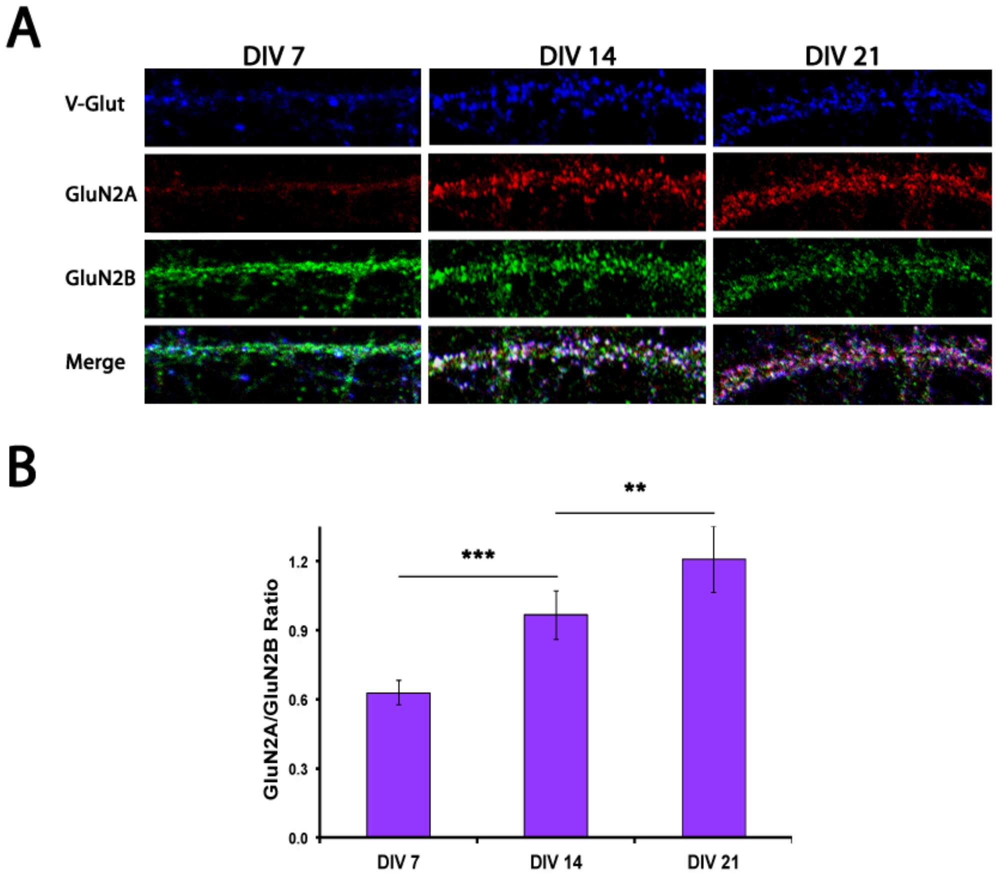

Figure S5. Synaptic GluN2A/GluN2B ratio increases during development in cultured hippocampal neurons

(A) Immunocytochemistry confocal images of DIV7, DIV14 and DIV21 hippocampal neurons stained for the vesicular glutamate transporter V-Glut 1 used as a synaptic marker (top, blue), GluN2A (second row, red) and GluN2B (third row, green), a merged image is displayed in the last row.

(B) Quantification of GluN2A/GluN2B ratio in V-Glut1 clusters over development. One-way ANOVA test was performed ($p<0.0001$), followed by Bonferroni *post hoc* test, *** indicates $p<0.001$ and ** $p<0.01$.

Supplementary methods

Immunocytochemistry

For immunocytochemistry displayed in Figure S5A, neurons were fixed for 10min in cold methanol (VWR) then washed twice with PBS. A blocking solution was next applied to avoid unspecific binding (PBS, 2% normal goat serum). The following primary antibodies were diluted in blocking solution and incubated for 2h at room temperature: mouse anti-GluN2A 1/100 (Zymed), rabbit anti-C-terminal-GluN2B 1/250 (Molecular probes), guinea pig anti VGlut1 1/500 (Chemicon). After washes in PBS, secondary antibodies (goat anti-rabbit Alexa 488, anti-mouse 546, anti-guinea pig 633, Invitrogen) diluted in blocking solution 1/1000 were incubated for 45min at room temperature. Coverslips were mounted in Prolong Gold (Invitrogen). Images were acquired on a Zeiss LSM510 confocal system using a 63X oil immersion objective (1.4NA). Alexa 488 was excited with an Argon laser at 488nm and detected through a bandpass filter (505-530nm). Alexa 546 was excited with a HeNe laser at 543nm and detected through a band-pass filter (565-615nm). Alexa 633 was excited with a HeNe laser at 633nm and detected through a band-pass filter (650-710nm). The intensity of labeling for GluN2A and GluN2B was measured in synaptic clusters, which were isolated using a sliding threshold (Lemieux et al., 2012) on VGlut1 co-staining.
